# An xception model based on residual attention mechanism for the classification of benign and malignant gastric ulcers

**DOI:** 10.1038/s41598-022-19639-x

**Published:** 2022-09-13

**Authors:** Yixin Liu, Lihang Zhang, Zezhou Hao, Ziyuan Yang, Shanjuan Wang, Xiaoguang Zhou, Qing Chang

**Affiliations:** 1 School of Health Science and Engineering, University of Shanghai for Science and Technology, Shanghai, 200093 China; 2 Department of Gastroenterology, Jiading Central Hospital Affiliated to Shanghai University of Medicine and Health Sciences, Shanghai, 201899 China; 3 School of Automation, Beijing University of Posts and Telecommunications, Beijing, 100082 China; 4 Institute of Digestive Surgery, Ruijin Hospital Affiliated to Shanghai Jiao Tong University School of Medicine, Shanghai, 200031 China

**Keywords:** Peptic ulcers, Biomedical engineering

## Abstract

To explore the application value of convolutional neural network combined with residual attention mechanism and Xception model for automatic classification of benign and malignant gastric ulcer lesions in common digestive endoscopy images under the condition of insufficient data. For the problems of uneven illumination and low resolution of endoscopic images, the original image is preprocessed by Sobel operator, etc. The algorithm model is implemented by Pytorch, and the preprocessed image is used as input data. The model is based on convolutional neural network for automatic classification and diagnosis of benign and malignant gastric ulcer lesions in small number of digestive endoscopy images. The accuracy, F1 score, sensitivity, specificity and precision of the Xception model improved by the residual attention module for the diagnosis of benign and malignant gastric ulcer lesions were 81.411%, 81.815%, 83.751%, 76.827% and 80.111%, respectively. The superposition of residual attention modules can effectively improve the feature learning ability of the model. The pretreatment of digestive endoscopy can remove the interference information on the digestive endoscopic image data extracted from the database, which is beneficial to the training of the model. The residual attention mechanism can effectively improve the classification effect of Xception convolutional neural network on benign and malignant lesions of gastric ulcer on common digestive endoscopic images.

## Introduction

Gastric cancer is a major malignant tumor that threatens human health and is one of the six most common cancers in the world. In China, gastric cancer is second only to lung cancer, ranking second in the incidence of cancer^1^. Gastrointestinal endoscopic screening in high-risk populations is still an effective means of early diagnosis and early treatment of gastric cancer. At present, how to improve the ability and level of early screening of gastrointestinal cancer in grass-roots hospitals is an urgent problem to be solved. In recent years, computer-assisted diagnosis (CAD) in medical imaging has received widespread attention for its important value in clinically assisted diagnosis and training^2^. The application approach of CAD in gastroscopic image-aided recognition is mainly based on the method of image recognition, that is, the initial network is trained by first extracting the relevant features of the image, such as color, texture, shape and spatial relationship, and then using the trained network to classify the lesion type of the corresponding lesion area. Pan et al. proposed that the F1 score of gastric cancer detection can reach 89.95% through the combination of feature fusion module and channel attention mechanism^3^. Sun et al. used a NonLocal mechanism to further enhance the neural network, which can improve the classification accuracy of benign and malignant data of gastric ulcer lesions to 96.79%^4^. Although the strategy of enhancing neural networks with attention mechanisms has improved the accuracy of CAD technology in the screening of gastroscopic lesion pictures, the effect of these optimization strategies in real application scenarios still needs to be further verified.

Establishing a tumor screening prevention and control system in primary medical institutions is an effective strategy to achieve early diagnosis and treatment of tumors. At present, the problems of a small number of gastroscopic screening cases and insufficient high-level experts in China’s grass-roots hospitals have put forward new challenges to the promotion and use of CAD in grass-roots hospitals.Residual attention mechanism is to embed the residual attention module into the model, and the residual structure, the soft mask branch, in the module strengthens the ability of the model to extract the good features of the picture^5^.

Thus, more valid information can be obtained on a single image, making CAD less dependent on large amounts of data during its application in primary hospitals. It is worth mentioning that residual attention mechanism improves the classification accuracy by stacking residual attention modules. But the excessive addition of residual attention modules is likely to make the neural network too deep, resulting in a gradient diffusion or explosion effect^6,7^, and finally making the model classification effect not rise but fall. Therefore, the optimal number of remaining attention modules to be added is evaluated in this study.

The improved residual attention mechanism is combined with the Xception model to classify and identify benign and malignant gastric ulcer lesions based on gastrointestinal endoscopic images in Shanghai Jiading Primary Hospital for a period of time. By comparing the changing characteristics of different model optimization strategies in the real application scenarios of grass-roots medical institutions, improvement plans are proposed, which provides new ideas for grass-roots hospitals to use CAD technology to carry out clinical research and promote the application of artificial intelligence.

## Methods

### Data sources

The original image dataset selected patients who were referred by a community health service center in Jiading District, Shanghai from January 2018 to November 2018 to the Digestive Endoscopy Center of Jiading Central Hospital for push-in digestive endoscopy, and take 2–6 gastroscopic images per patient. Then, the dataset that meets the requirements of image pretreatment will be divided into 2 groups according to the pathological results of patients, 109 cases in the benign group, aged 19–88 years, with an average age of 56.47 years, and 69 cases in the malignant group, aged 29–90 years, with an average age of 64.93 years. Finally, we collected a total of 819 gastroscopic images from 178 patients.

This study was approved by the Ethics Committee of Shanghai Jiading Central Hospital and is in line with the relevant statements of the Declaration of Helsinki, all methods were performed in accordance with the relevant guidelines and regulations, and all patients signed informed consent forms. The labeling of image data is done by two or more attending physicians of the Department of Gastroenterology using cross-labeling, and they are agreed through discussion when opinions differ.

### Data preprocessing

**Figure 1:**
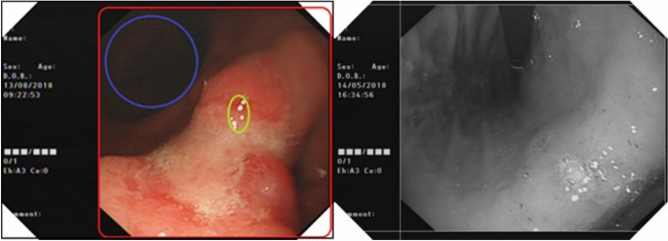
The original picture area division (left) and the area of interest boundary detection effect (right).

Endoscopic images, due to the particularity of their imaging methods, usually have problems such as uneven illumination and low resolution^8^, and if images are not preprocessed, it will affect the final analysis results of the network model. In this study, the endoscopic images collected and obtained were divided into two areas: the irrelevant information area and the region of interest, as shown on the left side of Fig. 1. The irrelevant information area, areas outside the red box, mainly contains the note information automatically generated by the device during the imaging process, such as time, device information, etc. The region of interest is the area of the tissue image, which contains dark areas, areas marked by blue circles, which contains dark areas that marked by blue circles, reflective areas that marked by yellow circles and target areas. Target areas include areas of normal tissue and areas of diseased tissue.

The dark area is caused by the low brightness of the auxiliary light source and the inaccurate position of the illumination when the camera is working, and the display of the area in the image is relatively dark and cannot provide accurate identification information. The reflective zone is a specular reflection produced by the residual liquid in the digestive tract, and the area is extremely bright and easily interferes with the doctor’s accurate judgment^9^.

In this study, the horizontal and vertical boundaries of the effective image area were detected by combining the Sobel operator and the Hough Transform line detection method^10^, as shown by the white thin line on the right side of Fig. 1. Then, the statistical characteristics of the dark and reflective regions are counted separately based on the HSV (Hue, Saturation, Value) color model^11^, the brightness threshold is set to V = 80 and the saturation threshold is S = 90.

**Figure 2:**
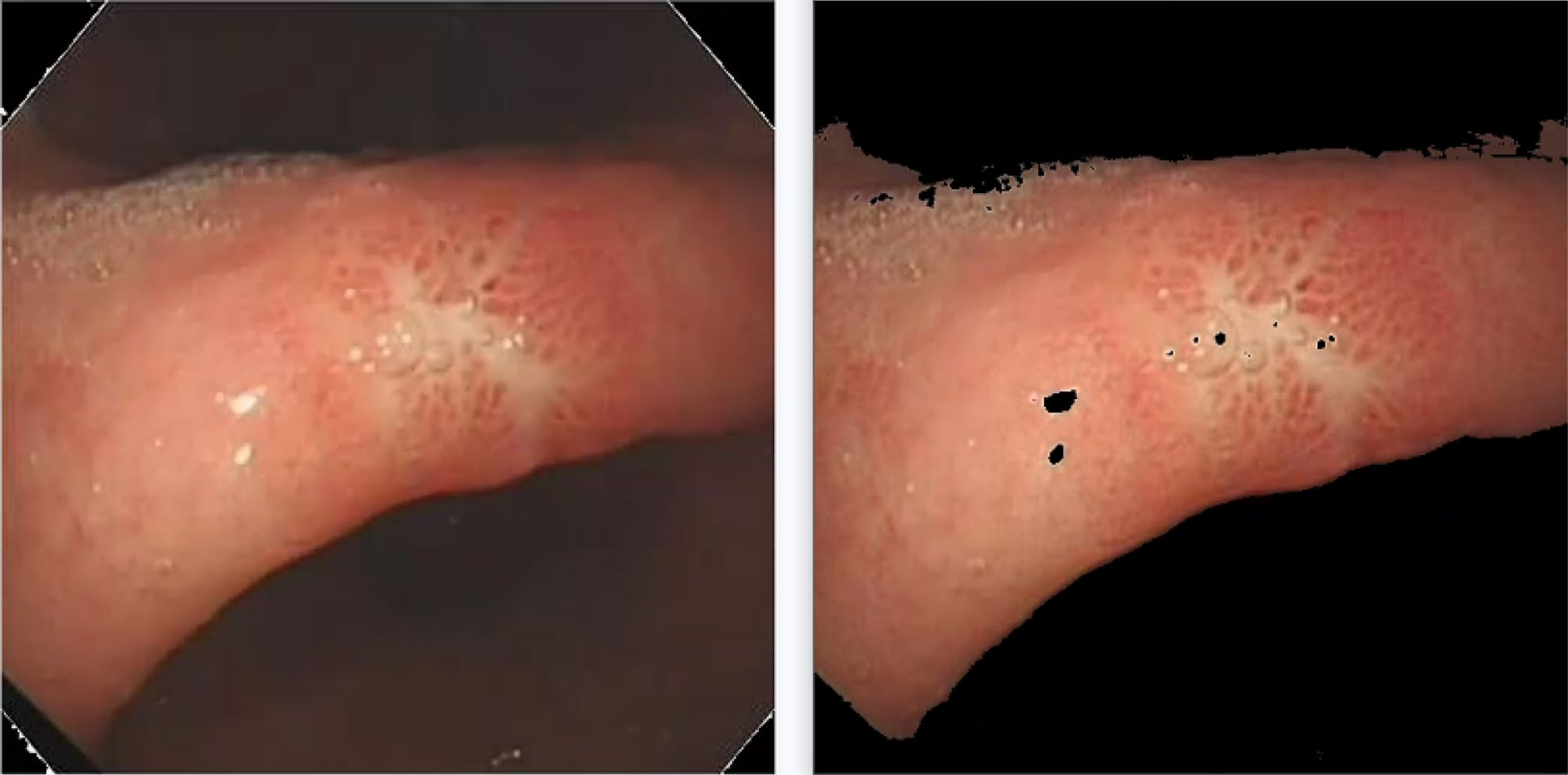
The original image (left) and the pre-processed (right) diagram.

Finally, according to the characteristics of regional clustering presented by the image, the discrete small areas are removed by means of multiple corrosion expansion cycle iterations. The final result of the pretreatment phase is shown in Fig. 2.

### Convolutional neural network

Convolutional neural network is one of the deep learning frameworks and is very good at solving problems in image classification. When building a convolutional neural network, convolution and pooling operations are usually used.

Convolution is used to extract high-dimensional and effective features from an image, while pooling is used to reduce the number of features and increase the robustness of the model. Finally, the joint output of the convolution and pooling layers can represent high-level features of an image^12^.

Among the convolutional neural network frameworks, Inception, ResNet, DenseNet and Xception are all classical convolutional neural networks.

Inception^13–15^ was first developed by the Google team in 2014 with the idea of improving the training efficiency of large networks. Inception aggregates information and effectively reduces the number of parameters by adding 1 $\times$ 1 convolutional kernels after 3x3 convolutional kernels, 5 $\times$ 5 convolutional kernels and 3 $\times$ 3 pooling kernels, which increases the width of the network and enhances its adaptability to scale.

ResNet^16^ won the ILSVRC classification challenge in 2015 and solved the problem of vanishing gradient caused by deep networks. ResNet enables deep networks to efficiently learn high-dimensional features of images by adding the output of previous layers to the output of this layer using shortcut connection.

Based on ResNet, DenseNet^17^ uses a more aggressive connectivity strategy where each layer concatenates the inputs of all previous layers and passes the output of each layer to all subsequent layers, which enhances the effect of image feature transfer between layers and allows the model to efficiently use features for image classification.

Xception^18^ introduces depthwise separable convolution and residual structure to achieve better performance than Inception, and makes more efficient use of the model parameters for the same number of parameters as Inception. Therefore, in our paper, we mainly use Xception to implement our model.

### Network model

In this study, Xception was used as the base network^18^. The Xception network is mainly composed of entry flow, middle flow, exit flow, depthwise separable convolution, etc., and its core is a depthwise separable convolution structure. Xception’s convolution and pooling through three flows, where the deep separable convolution that reduces the complexity of the network ensures the maximum information transmission between the layers in the network, and Xception widens the network at the same time. Finally, the performance of Xception network is improved while maintaining the same number of inception v3 parameters.

The improved Xception is based on the original Xception model, incorporating an improved residual attention mechanism to improve the network’s ability to extract global information. The model also uses 1*1 convolution to reduce data dimensions and reduce the amount of parameter calculation.

#### Xception module

The Xception module is stacked by a combination of three flow structures, each containing batch normalization (BN), rectified linear units (ReLu) activation functions, and depthwise separable convolutional kernel of 33. In the Xception module, the data is first processed by the entery flow, then through the middle flow, and finally further processed by the exit flow^18^. The 36 convolutional layers in the Xception architecture are constructed into 14 modules, all of which have linear residual connections except for the first and last ones^18^.

Since the output value and output distribution of each layer will change with the internal operation of each layer, the eigenvalues are standardized through BN to balance the distribution of the output of the neural network layer to solve the problem that the number of convolutional layers is too large to cause effective forward propagation. The ReLu activation function can improve the fitting ability of the network model because of its fast convergence speed, few calculation parameters and the operation rules also introduce nonlinear factors to the network model. Depthwise separable convolution with residual connections in a linear stack makes the model structure very easy to define and modify^18^.

**Figure 3:**
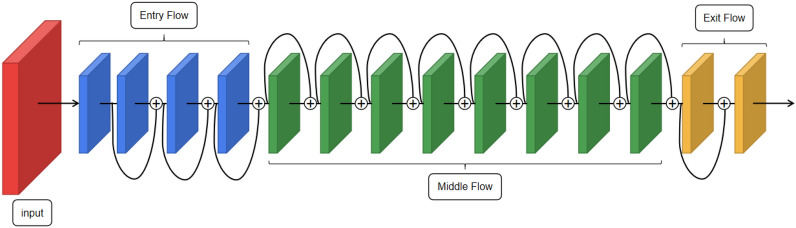
Xception module structure.

The Xception module structure is shown in Fig. 3.

The blocks in each flow consist of BN, ReLU activation function, 33 depthwise separable convolutional kernel and the max-pooling layer, while the residual structure of entry flow and exit flow consists of BN and 11 convolutional kernels.

#### Residual attention module

In a convolutional neural network, local connections are used between the adjacent layers of the network to acquire local characteristics of the image, and the receptive field of the upper-level network obtains the global information of the feature map by continuously superimposing the feature extraction layers. However, when the amount of image data is insufficient, it is difficult to ensure that the network model can capture a large number of features, and then the correct information cannot be learned. The residual attention mechanism further optimizes network model performance by preventing the use of false gradients to update parameters in the network, thereby enhancing meaningful features and inhibiting meaningless information^5,19^. It provides an effective solution for how convolutional neural networks can be trained in the absence of sufficient data^20^.

The residual attention module is divided into two branches: trunk branch (TB) and soft mask branch (SMB). TB is mainly used for feature processing, and its input *x* corresponds to the output *T*(*x*). While SMB mainly acts as a feature selector, and also acts as a filter during gradient updates. When the SMB acts as a filter, it enhances the robustness of the attention module and effectively reduces the effect of noise on the gradient update. The bottom-up and top-down structure is used in SMB to learn the mask *M*(*x*) of the same size, which facilitates the weighted output feature *T*(*x*)^5^.

The main formula for SMB is as follows:

1 $$\begin{aligned} \frac{\partial M(x, \theta ) T(x, \phi )}{\partial \phi }=M(x, \theta ) \frac{\partial T(x, \phi )}{\partial \phi } \end{aligned}$$

where the $\theta$ is a parameter for SMB and the $\phi$ is a parameter for TB.

The calculation rules after the combination of TB and SMB are as follows:

2 $$\begin{aligned} H_{i,c}(x)=(1+M_{i,c}(x))\times {F_{i,c}(x)} \end{aligned}$$

*x* is the input matrix. *F*(*x*) is a feature generated by a convolutional neural network in TB. *M*(*x*) is valued between [0, 1], when *M*(*x*) approximates zero, *H*(*x*) approximates the original feature *F*(*x*), therefore, in the residual attention module, SMB can act as a feature selector to enhance a good feature while suppressing noise in TB, at least it does not negatively affect the good original feature.

In our study, the block in the middle flow of the Xception network was used to replace the residual unit in the original residual attention module, which not only ensured that the feature map output size was unchanged, but also maximally optimized the feature.

**Figure 4:**
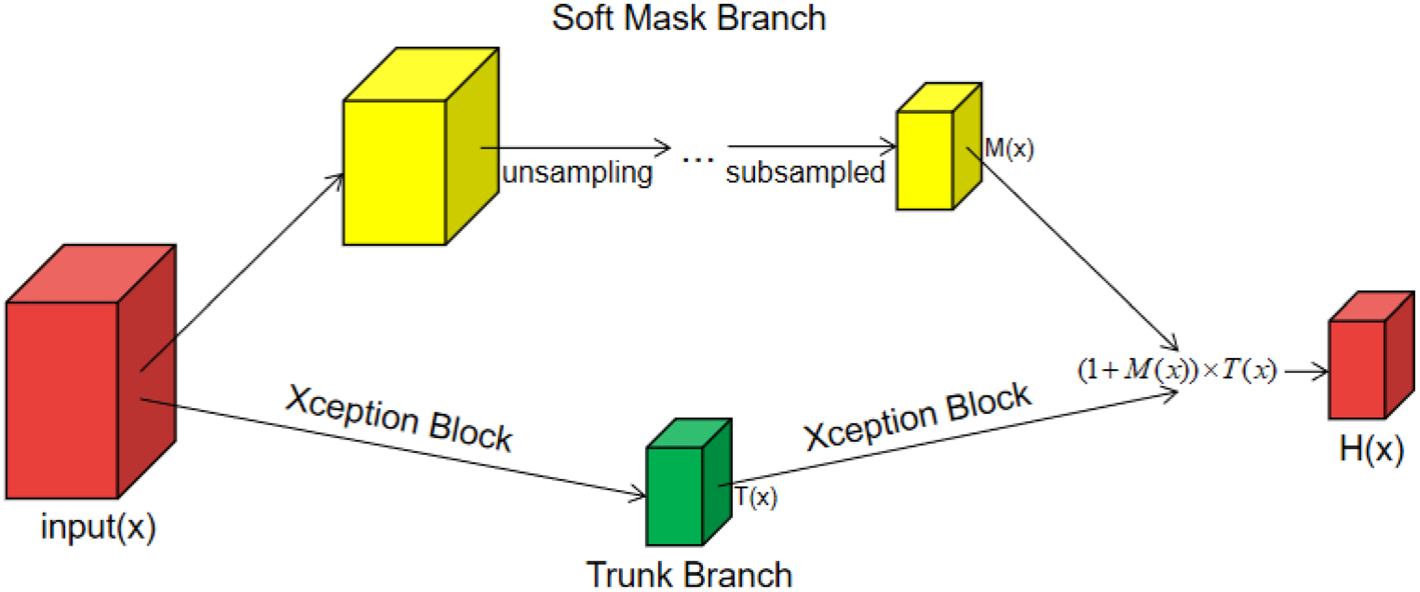
Residual attention module structure.

A schematic diagram of the residual attention module is shown in Fig. 4.

#### Overall architecture

In the improved Xception model, there are mainly three flow and three residual attention modules. The three flows are composed of 4, 8 and 2 Xception blocks respectively. Each block in the entry flow and exit flow consists of two convolutional layers of 33, containing depthwise separable convolutions, and a maximum pooling layer superimposed on each other. And the residual connection structure between blocks is composed of a convolutional layer of 11, while each block in the middle flow is composed of three depthwise separable convolutional layers of 33. The three residual attention modules contain 3, 2 and 1 subsampling and upsampling respectively, and the final subsampled feature map size of each module is consistent with the smallest feature map size in the entire network.

**Figure 5:**
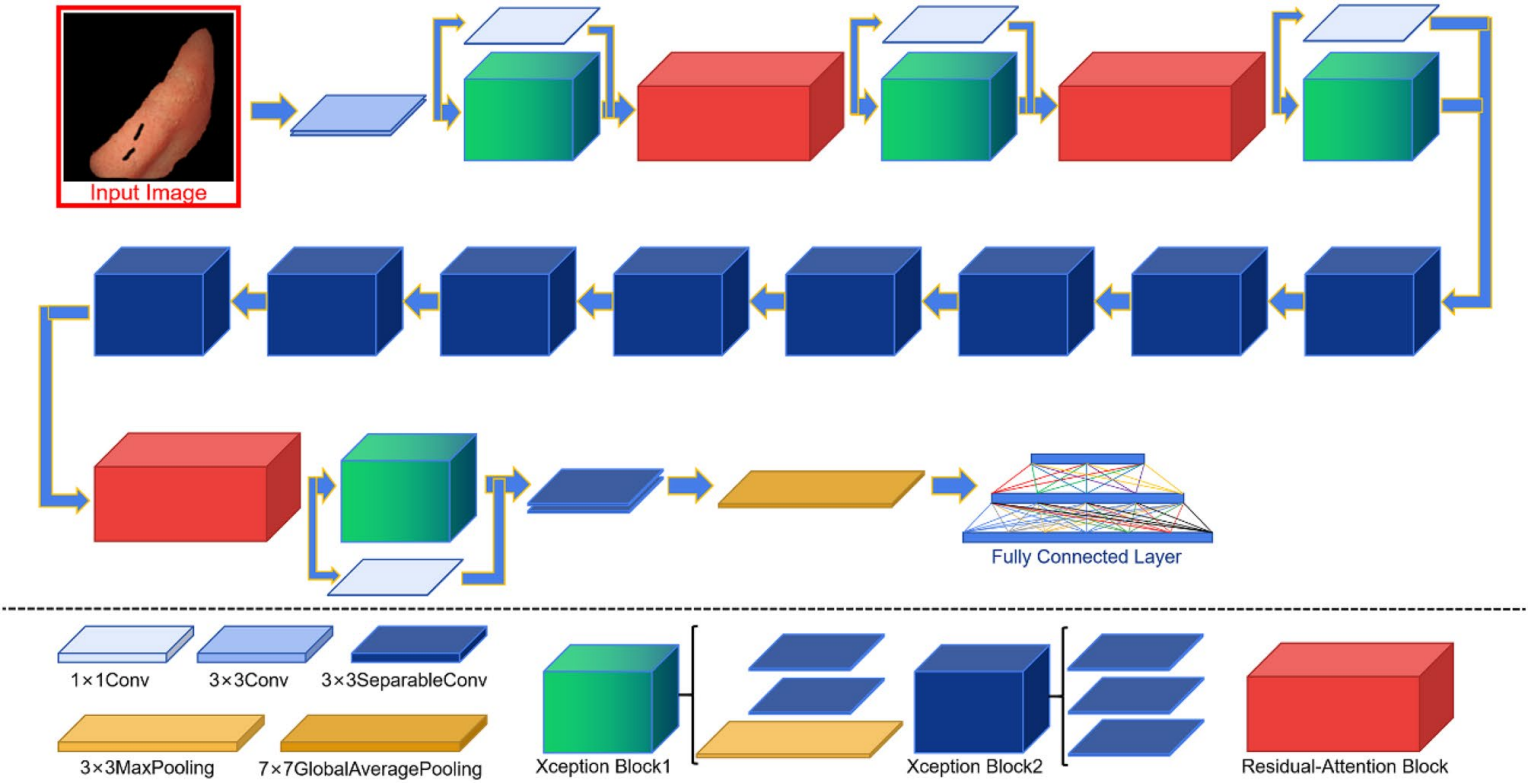
The overall network structure.

The residual attention module is embedded behind blocks 2, 3 and 12 of the Xception model. The main network structure is shown in Fig. 5.

### The problem of unbalanced sample distribution

#### Weighted loss

In order to solve the problem of uneven distribution of categories, weighted cross-entropy is used as the loss function^21^, and the weight value of the loss function of each category is set to improve the learning ability of the model to a small number of malignant ulcer samples.

The cross-entropy loss function is also known as log-likelihood loss. The formula for the ordinary cross-entropy loss function is:

3 $$\begin{aligned} loss(x_{class})=-\log {\dfrac{e^{x_{class}}}{\sum _je^{x_j}}}=-x_{class}+\log \left( \sum _je^{x_j}\right) \end{aligned}$$

The formula for the weighted cross-entropy loss function is:

4 $$\begin{aligned} loss(x_{class})=-W_{class}\times (-x_{class}+\log \left( \sum _je^{x_j}\right) ) \end{aligned}$$

#### Label shuffling

In our study, the proportion of data for each category in the obtained experimental dataset was uneven, so the class balancing strategy of label shuffling was adopted^22^. Its main principles are as follows:

(1). Define two lists, category lists and image lists of each category. (2). The initially defined image list is arranged in the order of labels, and the number of image samples of each category is counted, finally the number *N* of samples of the category with the largest number of image samples is obtained. (3). The remainder of the sample *n* of each type is taken from *N*, and the obtained remainder is used as the index value of the category, then the images are randomly extracted from each type to generate a list of images of this class. (4). The random list of all categories is connected together, and the final image list is obtained after reorganization, then the sequence in this list is used as the read-in sequence of the data for model training.

### Ethics approval and consent to participate

This study was approved by the Ethics Committee of Shanghai Jiading Central Hospital and is in line with the relevant statements of the Declaration of Helsinki, all methods were performed in accordance with the relevant guidelines and regulations, and all patients signed informed consent forms.

## Results

### Evaluation metrics

To verify the effectiveness of the method, the following metrics were selected: F1-score^23^, accuracy, precision, sensitivity, and specificity^24^. These indicators are widely used in the performance evaluation of medical image classification methods, for medical images, sensitivity indicates the probability of correct detection of lesions, and specificity indicates the probability of avoiding misdiagnosis. It is defined as follows:

$Accuracy=\dfrac{TP+TN}{TP+FP+TN+FN}$, refers to the proportion of the number of samples correctly classified by the model to the actual total number of samples.

$Precision=\dfrac{TP}{TP+FP}$, refers to the proportion of the number of samples judged positive by the model to the actual number of all positive samples.

$Sensitivity=\dfrac{TP}{TP+FN}$, refers to the probability of actually being sick and correctly judged as diseased according to the model screening standards.

$Specificity=\dfrac{TN}{FP+TN}$, refers to the probability that the actual disease-free is correctly judged as disease-free according to the model diagnostic criteria.

$F1-score=\dfrac{2\times {Sensitivity}\times {Precision}}{Sensitivity\times {Precision}}$, is defined as the harmonized average of precision and sensitivity, which can comprehensively evaluate the classification effect of the network model.

In the above equation, TP (True Positive) is the number of correctly classified positive samples, FN (False Negative) is the number of positive samples that were incorrectly judged negative by the model, TN (True Negative) is the number of negative samples that are correctly classified, FP (False Positive) is the number of negative samples that were incorrectly judged positive by the model.

### Experiments analysis

The computer CPU used in this experiment is Intel(R) Xeon(R) Silver 4210R with single-core, dual-threading and 2.4GHz clock speed. The graphics card is NVIDIA GeForce RTX 3070 with 8GB memory capacity. The Python version is 3.8.8, the Pytorch version is 1.9.0, the CUDA version is 11.1, and the cuDNN version is 8005. The initial learning rate is set to 0.02, and the weights of the neural network are updated by the Adam optimization algorithm during training. The momentum is 0.89, the decay rate is 5e−4, and the batch size is set to 8.

#### The effect of residual attention modules at different locations on the model

**Figure 6:**
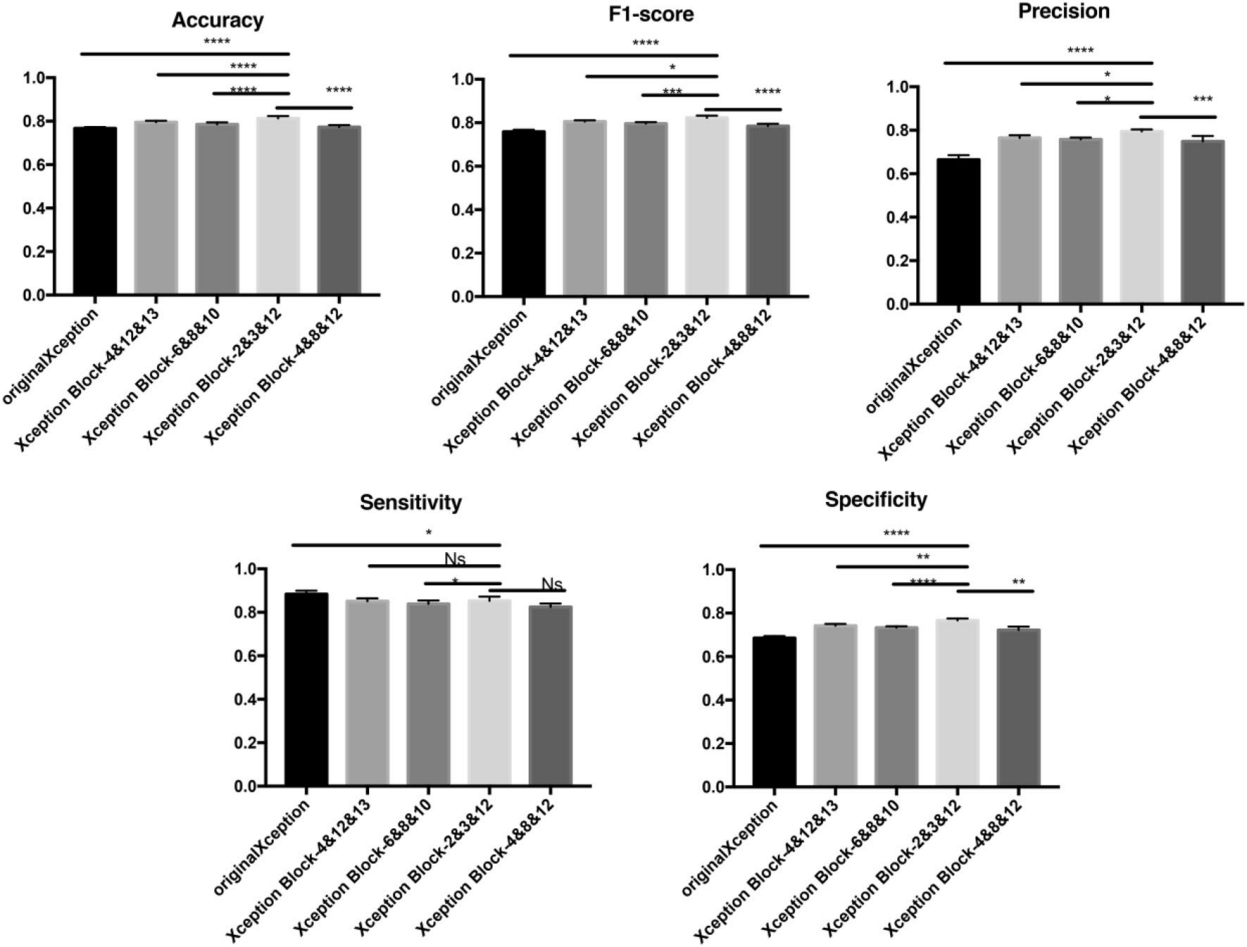
Statistical analysis of the influence of residual attention modules in different positions on the model.

Table 1 compares the addition of residual attention modules to different stages in the Xception network. It can be seen that the model obtains the optimal result when the residual attention module is added to the second, third and twelfth Xception modules, and the residual attention module is added mainly according to the change of feature map size. According to the flow division of Xception, the residual attention module is added to the fourth, twelfth and thirteenth Xception modules, the effect is not as good as after the second, third and twelfth Xception modules, which may be due to the fact that the feature map becomes very small after passing through the thirteenth Xception module, only 77 in size, which is not enough to provide accurate spatial information. The main reason for adding to the sixth, eighth and tenth Xception modules is that the middle flow in Xception is used to learn correlations and optimize features. Finally, adding the residual attention module to the fourth, eighth and twelfth Xception modules is followed by a comprehensive reference to Xception Block—4 &12 &13, Xception Block—6 &8 &10 and Xception Block—2 &3 &12, but it is found that the effect is very similar to Xception Block—6 &8 &10, and the effect is not as good as Xception Block—2 &3 &12. In general, it is found that adding a residual attention mechanism after the size change of the feature map can effectively improve the extraction of good features by the model, so as to achieve the purpose of enhancing the model effect.At the same time, we make statistical analysis on the results, and the statistical method selected was one-way anova analysis. The results are shown in Fig. 6.

**Table 1:** Effects of residual attention modules in different locations on the model.

Positions	Size	Accuracy	F1-score	Precision	Sensitivity	Specificity
originalXception	–	0.76993865	0.77012099	0.70000000	**0.85801445**	0.69948925
Xception block—4 &12 &13	14*14, 14*14, 7*7	0.79938650	0.80983488	0.77555556	0.84843030	0.75103426
Xception block—6 &8 &10	14*14, 14*14, 14*14	0.78650307	0.79627132	0.75666667	0.84126917	0.73376579
Xception block—2 &3 &12	55*55, 28*28, 14*14	**0.81411043**	**0.81815003**	**0.80111111**	0.83750914	**0.76826882**
Xception block—4 &8 &12	14*14, 14*14, 14*14	0.77361963	0.78482816	0.74777778	0.82624224	0.72161833

Significant values are in bold.

#### Classification effects of different models

**Figure 7:**
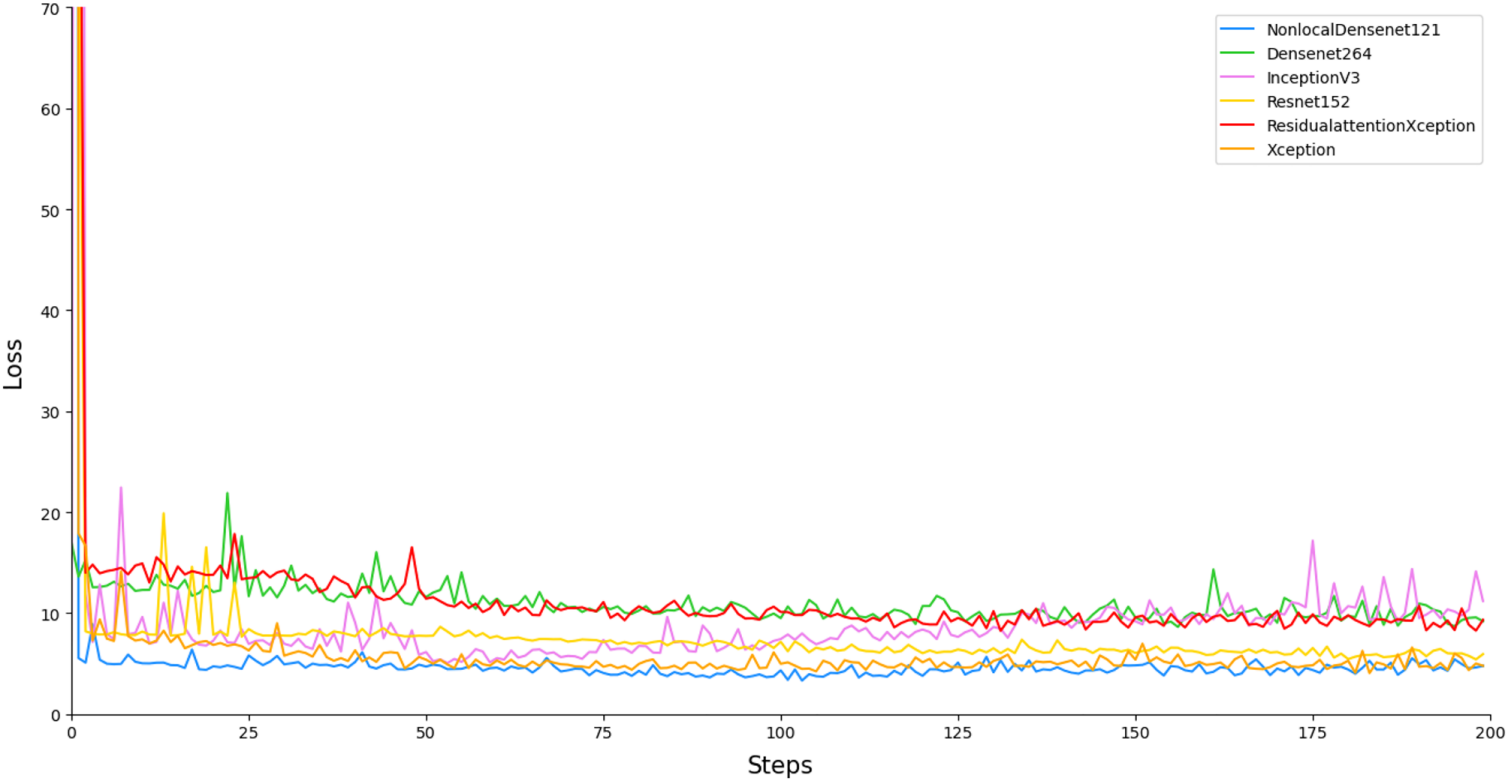
Loss function for each model.

**Figure 8:**
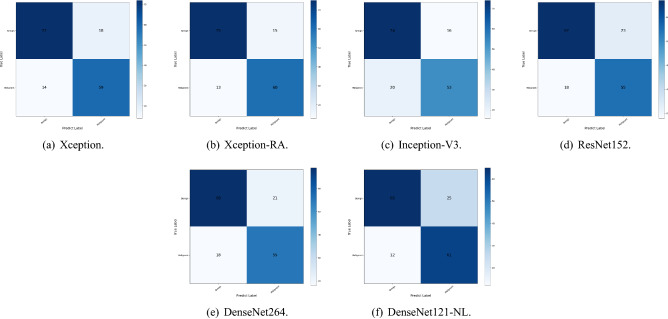
Confusion matrices of all comparison models.

The improved Xception model adds a residual attention module on the basis of the original Xception network to further enhance the model’s ability to extract good features and suppress meaningless information in real application scenarios with insufficient data, thus having better performance than other common convolutional neural networks in the same scenario. The results of the improved Xception compared with Xception, Inception-V3, ResNet152, DenseNet264 and NonLocal+DenseNet121 are shown in Table 2. The loss function convergences of all comparison models is shown in Fig. 7 and the confusion matrices^25^ of all comparison models is shown in Fig. 8.

**Table 2:** Comparison of results for different models.

Methods	Accuracy	F1-score	Precision	Sensitivity	Specificity
Xception-RA	**0.81411043**	**0.81815003**	**0.80111111**	**0.83750914**	**0.76826882**
Inception-V3	0.72024540	0.72120406	0.65777778	0.80174111	0.65531431
ResNet152	0.73190184	0.73948900	0.69000000	0.79866572	0.67278393
DenseNet264	0.73803681	0.75428927	0.73000000	0.78182436	0.69346031
DenseNet121-NL	0.74846626	0.76006952	0.72222222	0.80296779	0.69563232

Significant values are in bold.

The accuracy of the improved Xception model in the classification of benign and malignant gastric ulcer lesions was 81.411%, F1-score was 81.815%, sensitivity was 83.751%, specificity was 76.827%, and precision was 80.111%.

#### Performance of each model under different case counts

**Figure 9:**
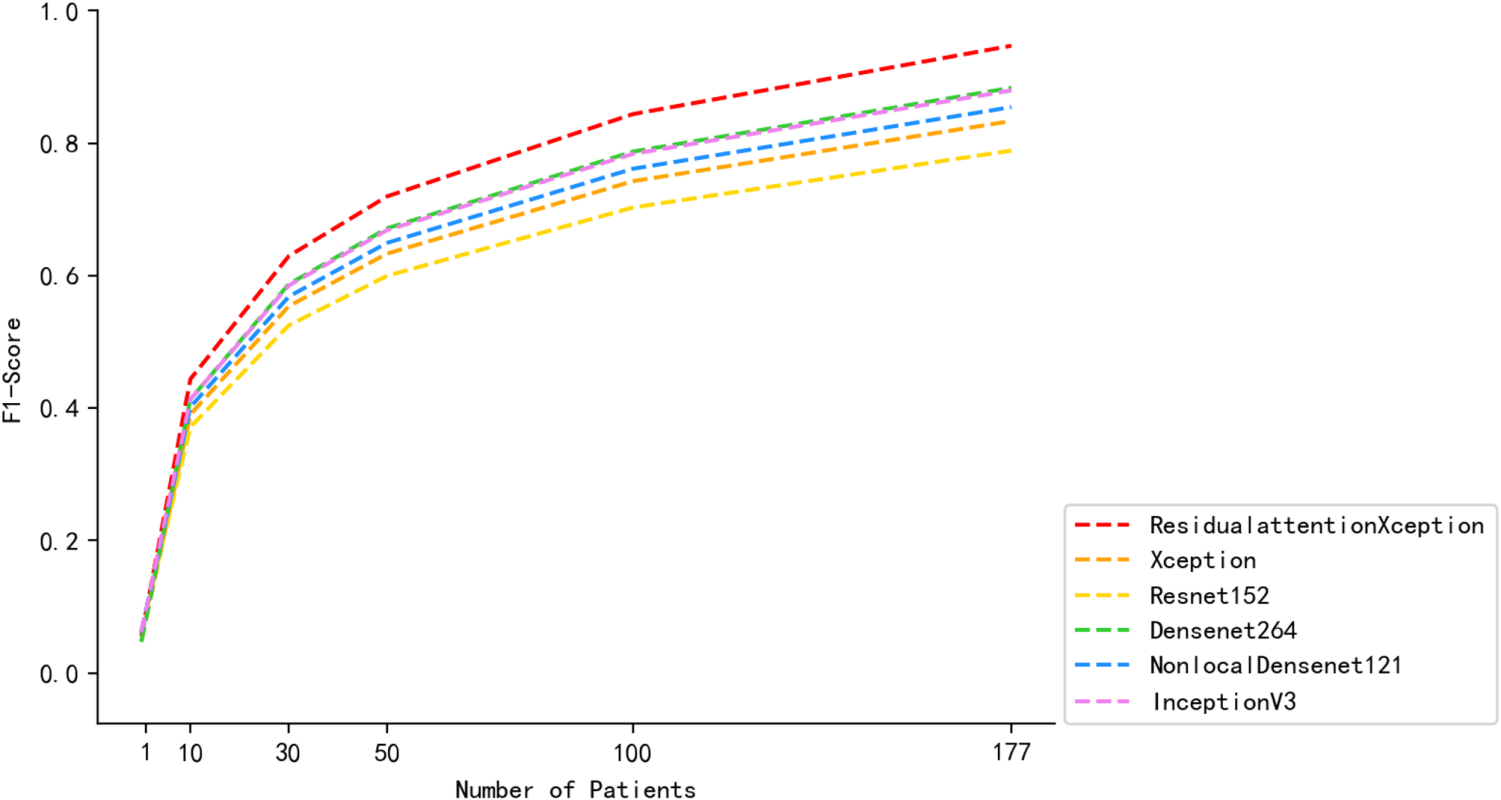
A graph of how the performance of the model varies with the number of medical records.

   As shown in Fig. 9, the model performance is different under different numbers of medical records, of which the overall performance of the improved Xception model in this paper is the best, the F1-score in 177 patients were observed to be 6.386% higher than those of second-placed Densenet264, and there is room for continuous improvement as the data increases.

It is undeniable that the increase in the amount of training data has a positive effect on the improvement of model performance^26^, but how to quickly apply the initial model when gastroscopic image data is scarce is a problem encountered in the promotion of artificial intelligence application and research in the gastroenterology department of primary hospitals. The improved Xception model in this paper can reduce the dependence on a large amount of data during model training, and provide a solution to the above problems.

#### External validation results

We collected gastroscopy data from some patients who visited the Gastroscopy Centre of Shanghai Jiading District Central Hospital in March 2022 as an external validation set, which contained 367 gastroscopy images from 100 patients. All images in the external validation set were preprocessed in the same way as the derivation dataset. We used the Xception-RA model, where the residual attention module was added after the second, third and twelfth Xception modules. The final results are shown in Table 3.

**Table 3:** External validation results.

Methods	Accuracy	F1-score	Precision	Sensitivity	Specificity
Xception-RA	0.80769231	0.82850836	0.78518519	0.88064991	0.73080277

#### Comparison of diagnostic results with endoscopists

As the diagnosis of benign and malignant gastric ulcers may vary depending on the characteristics of the lesion, we invited two experts in gastroscopy and clinical aspects from Shanghai Jiading District Central Hospital to participate in our study. The two expert doctors have 10 and 12 years of clinical experience respectively and both have been working in the gastroscopy centre for 7 years.

**Figure 10:**
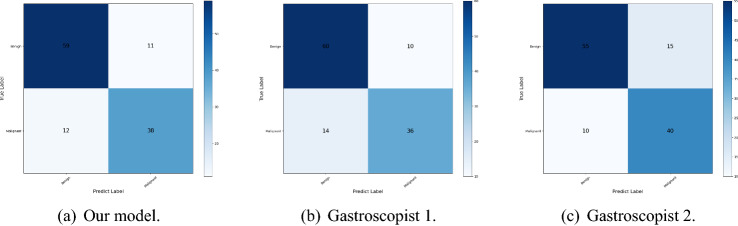
Diagnostic matrices.

We randomly selected 120 gastroscopic images from 50 patients to allow two gastroscopists and our model to make the diagnosis of benign and malignant gastric ulcers, the judgement process was carried out individually. The results are shown in Table 4 and the diagnostic matrices is shown in Fig. 10.

**Table 4:** Results of comparison with gastroscopists.

Participants	Accuracy	F1-score	Precision	Sensitivity	Specificity
Our model	**0.808**	**0.837**	0.831	0.843	0.760
Gastroscopist 1	0.800	0.833	0.811	**0.857**	0.720
Gastroscopist 2	0.792	0.815	**0.846**	0.786	**0.800**

Significant values are in bold.

As can be seen in Table 4, our model outperforms expert gastroscopists on the accuracy metric and the F1-score metric, and our model is very close to the optimal values for the precision metric, sensitivity metric, and specificity metric. Therefore, after discussion among the expert gastroscopists, they agreed that our model has better performance in classifying benign and malignant gastric ulcers.

## Discussion

In our study, the ability of the model to extract features on a single picture is enhanced by introducing the residual attention mechanism into the neural network model, thereby solving the problem of insufficient data available for training in primary hospitals. Using digital image processing methods such as Sobel operator and HSV color model, the problem of poor image quality caused by uneven illumination of fibre opic endoscope is preprocessed, which can eliminate the interference areas that are unrelated to the diseased image or are difficult to distinguish, and the residual attention mechanism is introduced to further strengthen the extraction and learning ability of Xception model for image features.

In the comparison stage of the model, the performance of the Xception-RA model was 6.564%, 5.808% and 7.264% higher than the second place in terms of accuracy, F1 score and specificity respectively, and the sensitivity also reached 83.751%, which was the best. Experiments have found that embedding the improved residual attention module behind the second, third and twelfth modules of Xception has better overall performance than other embedding methods, most likely because the change in feature map size allows the model to extract more good features, resulting in better classification ability. In addition, in the rapid application testing stage of the model, the learning ability of the Xception-RA model under the conditions of different number of cases is higher than that of other comparison models, and it can be further improved with the supplement of data.

Although we used the shortcut connection strategy, we found that as our model had already reached a very deep layer, further deepening the layers would cause vanishing gradient and exploding gradient, which would seriously affect the classification performance of the model. Therefore, we decided to try using the dense connection strategy to reduce the parameters of our model in the future, in order to continue to improve the performance of our model and effectively eliminate the vanishing gradient and exploding gradient problems.

## Conclusion

Gastroscopic screening is the main early diagnosis strategy for gastric cancer. Improving the gastroscopic screening capacity of primary hospitals is the key to gastric cancer screening in the community^27^. The introduction of CAD technology can make up for the lack of gastric cancer screening capacity in primary hospitals within a certain period of time. When CAD technology is introduced into medical institutions, it is necessary to use the existing data of the institution for training before it can meet the requirements of use. At present, because patients are more willing to go to grade A tertiary hospitals, the data available for training in primary hospitals is relatively insufficient, thus limiting the promotion and application of CAD technology in primary hospitals.

After our model learns a small amount of image data, it can well classify benign and malignant gastric ulcer lesions on gastrointestinal endoscopic images, which is conducive to the rapid deployment and application of the model in the primary hospital, and plays a certain auxiliary role in the early screening of gastric cancer. Our study can also provide new research ideas for primary medical institutions to deploy and promote new diagnostic and therapeutic technologies related to machine learning or deep learning.

## Data Availability

The data that support the findings of this study are available from Jiading Central Hospital Affiliated to Shanghai University of Medicine & Health Sciences, but restrictions apply to the availability of these data, which were used under license for the current study, and so are not publicly available. Data are however available from the corresponding authors upon reasonable request and with permission of Jiading Central Hospital Affiliated to Shanghai University of Medicine & Health Sciences.
